# Asymmetric Genome Organization in an RNA Virus Revealed via Graph-Theoretical Analysis of Tomographic Data

**DOI:** 10.1371/journal.pcbi.1004146

**Published:** 2015-03-20

**Authors:** James A. Geraets, Eric C. Dykeman, Peter G. Stockley, Neil A. Ranson, Reidun Twarock

**Affiliations:** 1 York Centre for Complex Systems Analysis, University of York, York, United Kingdom; 2 Astbury Centre for Structural Molecular Biology, University of Leeds, Leeds, United Kingdom; University of Texas at Austin, UNITED STATES

## Abstract

Cryo-electron microscopy permits 3-D structures of viral pathogens to be determined in remarkable detail. In particular, the protein containers encapsulating viral genomes have been determined to high resolution using symmetry averaging techniques that exploit the icosahedral architecture seen in many viruses. By contrast, structure determination of asymmetric components remains a challenge, and novel analysis methods are required to reveal such features and characterize their functional roles during infection. Motivated by the important, cooperative roles of viral genomes in the assembly of single-stranded RNA viruses, we have developed a new analysis method that reveals the asymmetric structural organization of viral genomes in proximity to the capsid in such viruses. The method uses geometric constraints on genome organization, formulated based on knowledge of icosahedrally-averaged reconstructions and the roles of the RNA-capsid protein contacts, to analyse cryo-electron tomographic data. We apply this method to the low-resolution tomographic data of a model virus and infer the unique asymmetric organization of its genome in contact with the protein shell of the capsid. This opens unprecedented opportunities to analyse viral genomes, revealing conserved structural features and mechanisms that can be targeted in antiviral drug design.

## Author Summary

Viruses are responsible for devastating illnesses in humans and cause significant commercial losses in livestock and crops. Their genetic material is transported into their host organisms via protein containers, called viral capsids, that act as Trojan horses: they release their cargo into the cells of their hosts, hijacking their molecular machinery for the production of progeny viruses. Imaging techniques exploiting the symmetric structures of viral capsids have been used to determine details of their organization to atomic resolution, opening up the possibility to design anti-viral agents against specific surface structures. In many viruses the genomes take on specific organizations as a consequence of their roles in capsid formation. In order to design an additional class of anti-viral drugs that interfere with this process, it is important to understand the asymmetric organization of the genome inside viral capsids. This is currently a challenge, as the averaging techniques used to achieve high resolution structures of the protein containers cannot be used. We present a new approach and demonstrate its predictive power here for a test virus. This paves the way for a better understanding of the functional roles of viral RNAs in virus assembly and their exploitation in anti-viral drug design.

## Introduction

Viruses are remarkable examples of symmetry and self-assembly at the nanoscale. The protein containers that encapsulate most viral genomes are formed from just a few different protein building blocks that self-assemble into particles with icosahedral symmetry, and can be described in terms of icosahedral surface lattices [1]. This geometry minimizes the amount of the genome fragment needed to code for the viral capsid, while maximizing its volume/surface area ratio; the principle of genetic economy [2]. Symmetry therefore plays a pivotal role in understanding virus structure. Symmetry averaging techniques have been used to determine viral capsid structures at atomic resolution by X-ray crystallography, and by reconstruction of such structures at medium resolution by cryo-electron microscopy (cryo-EM). However, not all viral components are organized with icosahedral symmetry. Cryo-EM can be used to refine such asymmetric structures provided that they are large enough in mass terms to contribute significantly to the image [3, 4].

However, asymmetric viral components normally contribute too weakly to the images obtained by cryo-EM to allow the refinement of an asymmetric model [5]. Note, in crystals of viral particles, the asymmetric features of the individual viruses usually do not dictate crystal packing contacts, and are therefore averaged out by the lattice. The important functional roles of such viral components in the viral life cycle are therefore difficult to characterize. An example is the single-copy of maturation protein (MP, also called A-protein) in bacteriophage MS2 that is hypothesized to replace a protein dimer in the capsid [6]. It attaches to the bacterial receptor during the infection to facilitate genome extraction. The asymmetric organization of the viral genome inside a capsid is also difficult to reconstruct. Indeed, MS2 is typical in that the high resolution crystal structure lacks density for the ∼3.7kb genome [7, 8], but cryo-EM reconstructions from both our group and others show extensive density for the RNA [6, 9–12]. This difference arises because of technical aspects of the ways the EM and X-ray data are collected.

We demonstrate here that a better understanding of the asymmetric organization of the viral genome within the capsid can be achieved if specifics about the contacts between capsid protein (CP) and the packaged genome are factored into an analysis of tomographic data. Recently we have shown that a number of positive-sense single-stranded (ss)RNA viruses encode dispersed, degenerate sequence/structure elements within their genomes that bind their cognate coat proteins specifically during assembly, facilitating capsid assembly efficiency [13–17]. These packaging signals (PSs) can have dramatic effects on the kinetics and fidelity of virion assembly [18]. There are widespread contacts between genomic RNA and capsid protein in picornaviruses, e.g. rhinovirus [19], and preliminary *in vivo* experiments for human parechovirus 1 suggest that they function as PSs (ongoing work with collaborators). The requirement for the PSs to contact the coat proteins of the viral capsid at specific positions in the capsid imposes a constraint on the conformation of the genome within each viral particle, that we are exploiting here to analyse tomograms of the packaged genomes.

In particular, we exploit knowledge of the PS positions with reference to the icosahedrally-averaged RNA cages that have been observed in a large number of viruses in proximity to capsid, to formulate constraints on the connections between the PSs. For example, if PSs are located at the vertices of these cages, as in the model system we are considering here, then the RNA organization in proximity to capsid can be modelled as connected paths along the edges of the RNA cage [15, 20]. If the majority of the potential binding sites are occupied by a PS in every particle, as is expected, for example, if such contacts are vital in triggering a conformational change in the protein building block with which they are in complex, then this path has the mathematical properties of a Hamiltonian path. In this paper, we will discuss explicitly an example for which the constraint set is given by Hamiltonian paths.

However, a similar approach can be adopted also for other viruses that violate some of the assumptions relating to our model system. For example, if PSs are stem-loops positioned along the edges of the polyhedral RNA cage, such as in Satellite Tobacco Mosaic Virus (STMV) [21], then constraints have to be formulated in terms of paths that permit edges to be transversed twice in opposite directions. The library of all possible paths with that property would then replace the library of Hamiltonian paths we are using for our model system here. Moreover, it is possible that only a fraction of the potential binding sites are occupied by PSs. For example, this might happen if PSs facilitate CP-CP interactions rather than CP quasi-conformer switching, as is the case for STNV [16, 22]. In this case, the constraint set corresponds to all paths on the polyhedral cage that connect subsets of the potential binding sites corresponding to the number of the PSs: these are therefore also not Hamiltonian paths. The overall strategy, however, would remain the same: deducing information from tomographic data using an appropriate constraint set formulated in terms of paths that encode information on the specifics of the RNA-CP contacts (PSs) and their positions relative to the ordered genome segments in the averaged structures.

Our previous modelling and the symmetry averaged structures of a large number of viruses from different families are consistent with the concept of such ordered genome segments in many viruses, including *Picornaviridae* [23], *Leviviridae* [9, 11], *Nodaviridae* [24], *Bromoviridae* [25, 26], *Tymoviridae* [27], *Comoviridae* [28] and satellite viruses [29–31]. Importantly, this asymmetric distribution of viral genomes within a virion may also be an essential factor in the extrusion/uncoating of these genomes as the first step in subsequent infection [6, 32–37]. The analysis presented here provides a novel way of deriving information on such asymmetric genome organizations, thus contributing to the understanding of such events.

Revealing RNA-protein contacts in molecular detail is a recent and novel challenge to our understanding of basic virus biology. In pursuit of this goal we recently used the association of MS2 phage particles to its natural receptor, a bacterial pilus, to create highly asymmetric complexes that could be subjected to asymmetric structure determination. This led to completion of a reconstruction using reduced (five-fold vs. icosahedral) symmetry averaging [12] and subsequently to a tomographic reconstruction of the whole virion using alignment and averaging of thousands of single particle tomograms [6]. The former result confirmed the presence of extensive RNA density, and the latter revealed its asymmetric structure; a first for any ssRNA virus. This suggests that the MP occupies a two-fold position in the otherwise icosahedral coat protein lattice, presumably replacing the normal CP dimer at that site. Unfortunately, the resolution of the asymmetric tomographic reconstruction is very low (39Å) and the molecular details are still unclear.

It is therefore important to develop new analysis techniques that are able to reveal such genome organizations based on a range of data from different techniques, including the low resolution information contained in tomographic data. We introduce here a new method that uses information from icosahedrally-averaged maps, as well as knowledge of the contact sites between genomic RNA and CP to analyse the low resolution, tomographic density maps via a constraint optimization technique revealing the putative asymmetric genome organization of bacteriophage MS2. As we describe in detail here, the constraint set for the analysis of MS2 is derived from circular Hamiltonian paths connecting the PS contact sites, and similar constraints are likely to apply also to other *Leviviridae* [9]. For other viruses, in which occupation of the majority of the PS binding sites is likely due to their function in assembly, and for which the PSs are positioned at the vertices of the RNA cage corresponding to the icosahedrally-averaged map of the genome in proximity to capsid, the constraint set is also given by Hamiltonian paths. However, the set of Hamiltonian paths would be distinct from the one used for our model system if the numbers of binding sites and the connectivity between them differ. We are providing detailed instructions on how to modify our code (freely available at http://hprna.github.io/) to accommodate such alterations. If there is evidence that the 5′ and 3′ ends are in proximity in the packaged genome as in our model system, then the set of constraints can be reduced to only the circular Hamiltonian paths; otherwise, the full set of Hamiltonian paths has to be taken into account. Our code includes a setting that allows switching between these options, to compute either circular or non-circular Hamiltonian path constraint sets as required. Note that this method also applies if some of the potential binding sites remain unoccupied in random positions across the ensemble of particles used to generate the tomographic data, as such random mistakes would not be reinforced during averaging over different particles: hence it is sufficient that the majority of PS binding sites are occupied. Note that in the case of insufficient information being available to decide a priori between multiple constraint sets (stemming from different assumptions on the specifics of the PS-mediated assembly scenario), the tomogram could also be interrogated against the different possible options. This could give an indication, perhaps in combination with additional experimental insights, as to which of the proposed mechanisms is most likely to occur.

The main purpose of this paper is to demonstrate that the method of using constraint sets, inspired by insights into the roles of PSs, can indeed result in a better understanding of tomographic data, and perhaps even reveal the asymmetric organization of the packaged genome, as in the example discussed here. In order to demonstrate this for a model system, the specifics of that system must be built into the formulation of the constraint set. However, as we argue above, the method of interrogating tomographic data via constraint sets inspired by PS-mediated assembly mechanisms is more generally applicable to wider classes of viruses.

## Results

### Geometric constraints on genome organization

**Fig 1:**
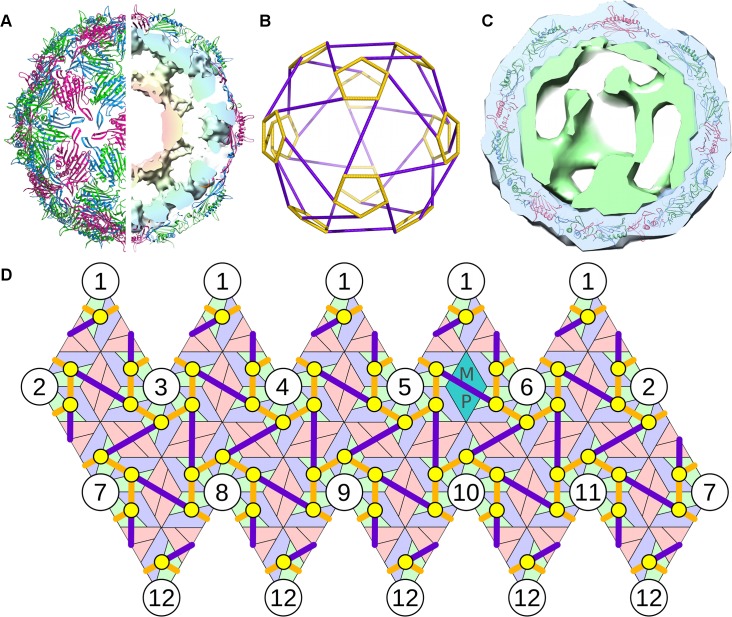
The model system—bacteriophage MS2. (A) The viral capsid is formed from 60 asymmetric and 29 symmetric copies of the CP dimers, with one MP that takes the place of a symmetric dimer (PDBID 2MS2). The genomic RNA is organized inside the particles in two shells, with the outer shell adopting the shape of a polyhedral cage in icosahedrally-averaged reconstructions. (B) Depiction of the polyhedral cage, showing long (purple) and short (orange) PS-PS RNA connections. (C) Asymmetrically averaged tomogram of bacteriophage MS2 bound to its receptor, the bacterial F-pilus. The portion of the electron density corresponding to the CP shell (and bacterial pilus) is shown in blue; green depicts the density for genomic RNA (and presumably some elements of the MP), which forms the basis for the analysis described in this study. The RNA density forms a shell that is intimately associated with the inside surface of the capsid. (D) A planar representation of protein container and polyhedral RNA organization, showing the relative positions of the 60 polyhedral vertices (PS positions, indicated as yellow circles) in contact with the 60 asymmetric CP dimers.

We illustrate this procedure here for the model system bacteriophage MS2. MS2 has a quasi-equivalent T = 3 capsid formed from 89 non-covalent CP dimers, comprising 29 symmetric ones (C/C) located at the particle two-fold axes, and 60 asymmetric ones (A/B) organized in groups of five around the capsid five-fold axes, and one MP that replaces a C/C dimer, see Fig. 1A. RNA PSs in the genome have been shown to act as allosteric regulators of the CP-dimer conformation, PS binding favouring formation of the A/B dimer [38, 39]. Thus, in an ideal case, we would expect to find 60 PSs within the genome. PSs are highly degenerate in nucleotide sequence. We have identified all the PSs in both MS2 and the related phage GA via a new analysis method based on biochemical RNA-CP binding and SELEX data [15].

In the icosahedrally-averaged MS2 cryo-EM map [11] the ∼3.7 kb long RNA genome appears inside the capsid as two concentric shells with density connections at the particle five-fold axes. This arrangement reflects the contacts that the genomic RNA makes between PSs and the CP layer, which appear as the outer shell, whilst the inner shell is the consequence of RNA segments that do not bind to CPs but extend into the interior of the capsid. The start and end points of these segments are located at the same five-fold vertex in the capsid [11]. Therefore, every PS is connected to two other PSs in the outer RNA shell, and hence the RNA in the outer shell, i.e. disregarding fragments extended into the interior, forms a connected path. If the path were disconnected, PSs at different five-fold vertices would have to be connected directly via RNA in the capsid interior, which is not consistent with the cryo-EM analysis in Toropova et al. [11]. The averaged outer shell density (Fig. 1A) is in the form of a polyhedral cage (Fig. 1B), positioned such that its vertices are in contact with the 60 asymmetric dimers (see yellow circles in the contact map in Fig. 1D). The RNA outer shell is intimately associated with the inside surface of the CP shell, as is also seen in the asymmetric reconstruction (Fig. 1C). The positions of the PSs in the genome determined earlier [15] suggest that the connections between PSs are single-stranded. The connected path described by the RNA in the outer shell is therefore a Hamiltonian path on that polyhedral RNA shell, i.e. a path that meets all vertices (aka PS positions). In particular, we determined all possible ways in which the RNA can be positioned in the icosahedrally-averaged density of the outer shell by computing all possible Hamiltonian paths on the polyhedron in Fig. 1B. Note that for viruses with different polyhedral RNA organizations the same method can be applied by computation of the Hamiltonian paths on the corresponding polyhedral density. Moreover, since Hamiltonian path computations only depend on the topology of the polyhedron, i.e. the network of connections between vertices irrespective of the lengths and orientations of the edges, the same library of Hamiltonian paths can be used for wider classes of viruses, such as those studied by van den Worm et al. [9] or bacteriophage GA [15].

In the case of bacteriophage MS2, additional biochemical information showed that regions close to the 5′ and 3′ ends of the genomic RNA were bound to the MP [40], which was positioned adjacent to one of the particle five-fold axes, replacing one of the CP dimers on a two-fold axis in the protein shell. This circularization reduces the number of possible Hamiltonian paths for the RNA. In particular, filtering out all those Hamiltonian paths with end points at the same five-fold axis, reduced the number to only 66 [20]. Since abstract paths have no directionality to them, each could potentially be realized by the RNA in two different ways by interchanging the positions of 5′ and 3′ ends, resulting in 132 path solutions. Since the resolution of the averaged tomogram, obtained via alignment and averaging of individual tomograms, was not sufficient to unambiguously identify the location of the MP, and the binding sites of the RNA were difficult to identify, we bookmarked all paths which started and finished within the eight five-fold axes closest to MP. This was a very conservative overestimate, which ensured that no possible path was missed in our analysis. Each of these (Hamiltonian) paths could potentially start at any of the five-fold vertices. In total, we therefore obtained a library of 8*5*132 = 5280 possible paths for the genomic RNA in the outer RNA shell. As mentioned above, this library can be applied to a wide range of RNA viruses, covering all those with a polyhedral RNA organization topologically equivalent to that of MS2.

The polyhedron describing the averaged density was given in terms of two types of edges (cf. Fig. 1B&D), 60 short and 30 long ones, and it had 60 vertices (cf. yellow circles in Fig. 1D). Each path in the library was therefore given as a sequence of 60 edges on the polyhedral shell, which were a mixture of short and long edges depending on the path. Each path provided information on which edges are simultaneously occupied or unoccupied, and hence correlated occupancy information on different edges.

### Analysis of the tomogram via graph theory

The library of putative path organizations was used as a set of constraints in the analysis of the asymmetric electron density for the outer RNA shell, which we isolated from the tomogram as described in Methods. Note that any path in the library provided information on which edges were likely to be occupied, given that occupation of some of the edges—or the lack thereof—could be confirmed based on the tomogram. The first step was therefore to determine a subset of the 90 edges of the averaged map (with reference to the polyhedron in Fig. 1B) that were likely occupied or unoccupied given the density distribution of the tomogram. We excluded all short edges as they were too short to distinguish unambiguously whether density represented the RNA-CP contact (i.e. PS) positioned at the vertex, or a connection between two PSs along a short edge. We moreover disregarded the five long edges (see S1 Fig) around the MP, as it was not possible to ascertain whether density in these regions arose from the MP, genomic RNA, or a combination of both.

**Fig 2:**
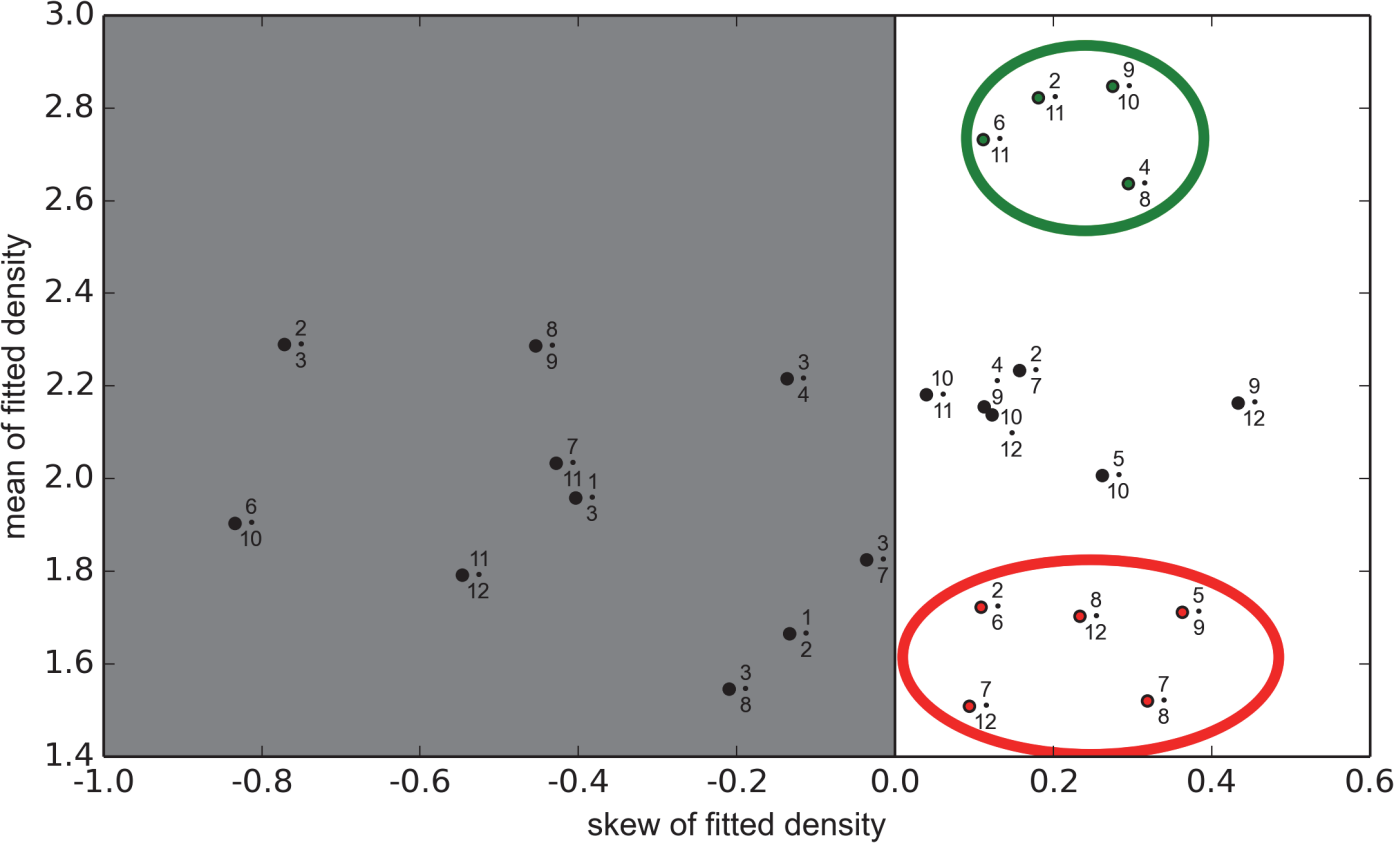
Classification of polyhedral edges as occupied and non-occupied. A comparison of the density profiles of the sampled long edge connections. The mean of a fitted normal distribution (y-axis) is scattered with a skewness parameter (x-axis). Connections with negative skew are disregarded as no statement about occupancy can be deduced in this case. From the remainder, two groups of four and five connections are identified as occupied (in the green circle) and non-occupied (red circle), respectively. These are used as constraints in the analysis.

As discussed in Methods, we attributed tomographic density to each of the 25 long edges of the polyhedral cage representing the icosahedrally-averaged density considered in this analysis and fitted it to a normal distribution. A ranking of the level of density associated with these edges was achieved using the mean of the fitted normal distribution. This method was used because outliers in the noisy, sparse dataset had less influence on the mean of the fitted distribution than they did with a simple arithmetic mean. Using the fitted mean, four connections stood apart from the others, with mean densities of 2.6–2.9, see Fig. 2, suggesting that these four edge connections were likely occupied by RNA in the virion. These were denoted as “occupied” connections, and were used as constraints in the analysis of the asymmetric structure.

To determine which connections could be classed “unoccupied”, we used the skew parameter of the sampled distributions to examine smearing of density. Skewness characterizes the balance of a distribution to either side of the peak density. As expected, the group of connections classed “occupied” above had a skew between 0.1–0.3. Negatively skewed connections were disregarded from the analysis, because a negative skew meant that there were only a very limited number of high-density points, which made up the cumulative density. Because of their low copy numbers, small fluctuations in sampling made a big difference to the overall density, and we therefore did not want to make a judgement of occupancy based upon these data. Using the skew parameter, the remaining data were therefore separated into distinct groups. The five data points shown in the red circle in Fig. 2, with mean values between 1.5–1.8, were adjudged “non-occupied”, i.e. characterized by an absence of density corresponding to RNA.

There were thus nine constraints on RNA organization that were used to compare the asymmetric structure with the library of all possible Hamiltonian path organizations: four long edges were deemed occupied, and five non-occupied.

### Constraint optimization yields RNA organization in proximity to capsid

**Fig 3:**
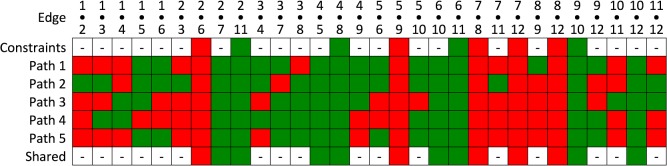
Constraints on the RNA organization consistent with the tomogram. Each possible RNA organization is characterized by which long edges (Fig. 1B, purple edges) are occupied in the polyhedral shell of the icosahedrally-averaged density. Long connections are labelled by the numbers of the five-fold vertices (Fig. 1D) they connect (x•y connecting five-fold vertices x and y). Constraints imposed in the analysis are indicated in the first row, with green indicating an occupied edge, and red an unoccupied edge. The five paths meeting these constraints are characterized according to occupied and non-occupied edges. The last row shows edges shared by all five paths.

Only five members of the library of all possible Hamiltonian paths were consistent with these nine constraints. In Fig. 3 we display the occupation of long edges with reference to the two five-fold vertices they connect, following the numbering scheme of vertices given in Fig. 1D. Note that the paths match for 13 of the 30 long edges, suggesting that the structure common to all paths is likely to be a prevalent feature in different viral particles.

**Fig 4:**
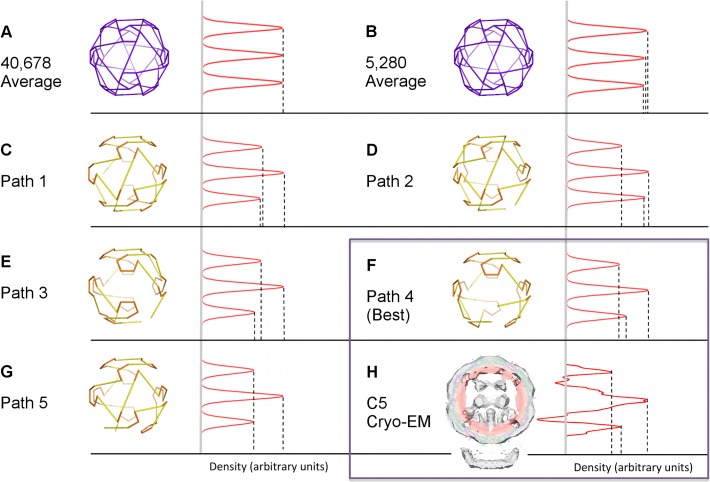
Symmetry averaging identifies Path 4 as the correct solution. C5-averaged densities in 1-D projection for tomographic data and the path solutions listed in Fig. 3 are compared. The vertical axis shows the radial distance from the centre of the capsid in angstrom, and the horizontal axis corresponds to the C5-averaged density at that radial distance in arbitrary units; density profiles for tomogram and path solutions are normalized by equalizing the maximum densities. Density profiles are shown for: (A) the average of all possible 40,678 Hamiltonian paths; (B) the average of all paths consistent with RNA interaction with the MP; (C-G) the density profiles for the five paths in Fig. 3 individually; (H) the C5 cryo-EM reconstruction from the tomogram, adapted from [20]. Path 4 (cf. Fig. 5A), identical to Path 3 (Fig. 5B) from a geometric point of view but positioned differently within the density with respect to MP, provides the closest fit with the cryo-EM data.

**Fig 5:**
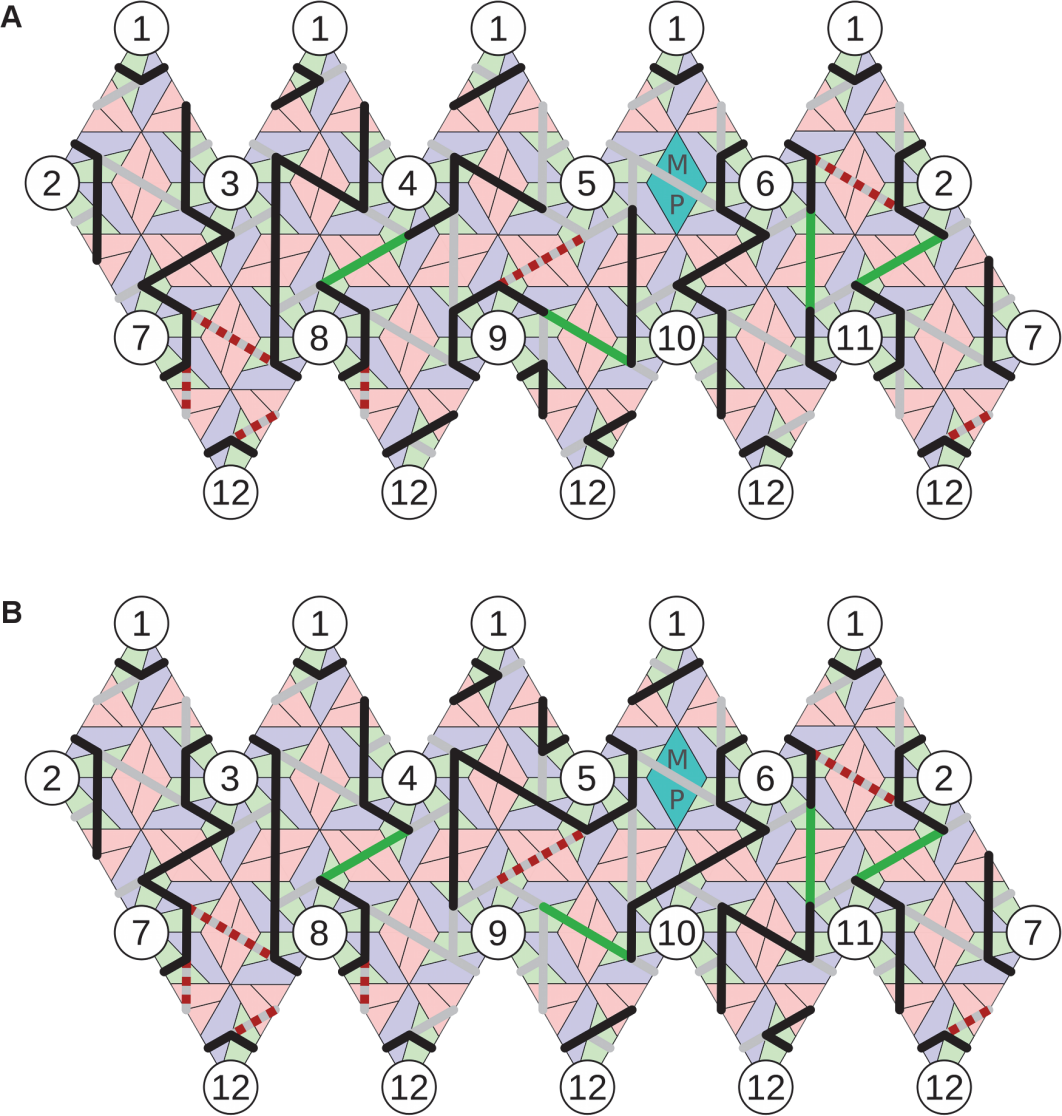
Hamiltonian path solution identified by the method. (A) The best match with the C5 averaged data (Path 4) starts and finishes at vertex 5 adjacent to the MP (cyan). Following the colouring convention in Fig. 3, red dashed lines show unoccupied and green lines occupied constraints; other occupied connections implied by our analysis are shown in black. The position of TR, the strongest PS, is denoted in yellow [15]; heterodimers are coloured in green-blue and homodimers in pink. (B) An alternative embedding of the same (geometric) path with a different orientation relative to MP. The path (Path 3) starts and finishes at vertex 9; hence the occupation of the connections differs from Path 4 in (A), even though the overall geometry of the path is the same.

Each path was a roadmap of connectivity between RNA-CP contacts. In order to decide if any of these putative RNA organizations was more likely to occur than another, we used the following criterion: We associated with each option a density distribution by ascribing density to occupied edges in proportion to their lengths and computed the density obtained by averaging around the five-fold axis adjacent to MP. We used this as a characteristic to benchmark against the five-fold averaged density determined experimentally [12] (Fig. 4H, adapted from [20]). Path 4 (Fig. 5A) closely matched (Fig. 4F) this distribution, whereas the other paths did not. This strongly suggested that Path 4 was indeed the correct model for the organization of the RNA in MS2. Remarkably, Path 4 is also consistent with results of two independent studies: the assembly pathways determined via kinetic modelling of capsid self-assembly [20], and the PS positions identified via a bioinformatics analysis of RNA SELEX data [15]. Our analysis here represents a completely independent reconfirmation that the organization of the viral genome in proximity to capsid is highly constrained and likely identical in every viral particle.

## Discussion

The analysis method introduced here has for the first time identified the conformational path taken by a viral genome in proximity to its capsid from the low-resolution density map of an asymmetric, averaged tomogram. Previously, a model of the asymmetric genome organization in the plant satellite virus STMV has been built [41]. That work relied on the icosahedrally-averaged crystal structure which revealed ∼70% of the viral genome to be in contact with the protein shell via a series of dsRNA segments ∼9 bp long [30, 42, 43]. The X-ray structure provided the first definition of RNA PSs [21]. In addition to the X-ray density the modelling used predictions of the most likely secondary structure elements within the genome to identify the sequences forming the double-stranded segments [44]. Ours is the first direct analysis of an asymmetric map containing RNA density. The method introduced here can be used to analyse any asymmetric dataset of a viral genome organization, provided that a distinct shell of density is seen in proximity to capsid in the averaged cryo-EM density, the contact sites between genomic RNA and capsid protein are known, and information regarding their positions and function can be used to formulate a constraint set on the connectivity between the PSs. Insights into PSs are becoming available for a number of ssRNA viruses via the use of CLIP-SEQ techniques [45]. In addition, there is a growing body of work directed at obtaining asymmetric structures for this class of viruses in order to understand how their genomes are released during infection. Our approach is therefore likely to provide important insights into genome organization in wider groups of RNA viruses. In particular, many RNA viruses show order in the organizations of their genomes in icosahedrally-averaged cryo-EM and X-ray structures [46], for example Bean Pod Mottle Virus [47], STMV [30] and Pariacoto virus [48]. In such cases, constraint sets in terms of paths with appropriate combinatorial properties can be used to map the putative asymmetric organization of their genomes into the corresponding symmetrically averaged densities and hence provide information on connectivity between the RNA-CP contact sites.

A better understanding of the asymmetric organization of viral genomes is vital if we are to properly understand the functional roles of genomes in RNA viruses. Recent research has revealed that far from being a passenger in the assembly of the viral particle, genomes critically enhance the efficiency of virus assembly via multiple dispersed, sequence-specific contacts with capsid protein [14]. These PSs act collectively in a cooperative manner [18, 49], and their relative placement in the tertiary structure of the genome is important for their function. In particular, it is the relative affinities of the PSs for CP at defined positions in the packaged genome that impact on the geometries of the assembly intermediates, i.e. on the structures of the partially assembled protein shells on pathway to capsid. For the virus discussed here, it had previously been shown that this interplay of PS affinities and capsid geometry results in a highly ordered genome organization in proximity to capsid. It has moreover been established that the same overall organization of the packaged genome occurs in an evolutionarily related virus, GA [9, 15], suggesting that there is a selective advantage for a specific genome organization in this family of viruses. This advantage can be explained in terms of assembly pathways: since PSs are instrumental in recruiting CP to the growing nucleus during PS-mediated self-assembly, the positions of the PS-CP contacts impact on the geometry of the assembly intermediates and hence on the assembly pathways. For the conserved genome organization identified in MS2 and GA earlier [15], this corresponds to an assembly pathway through the most stable intermediates, i.e. those forming a maximal number of CP-CP bonds [20].

This example illustrates that structural information on genome organization obtained via the method introduced here has important implications for our understanding of the functional roles of viral genomes in virus assembly. More broadly, the method applies to any virus for which RNA-protein contacts are important for virus assembly, i.e. all viruses that follow a PS-mediated assembly process [14]. PSs are known to exist in a number of viral families including those infecting humans, e.g. alphaviruses [50], and plants [51], so this method is applicable to wider groups of RNA viruses. We note that the exact mechanism by which PSs act to enhance virus assembly can vary. For example, for MS2 the PS-CP contacts trigger an allosteric switch between the two types of protein building blocks required for productive capsid formation, while for STNV PSs promote formation of the coat protein capsomere [22], a trimer, by overcoming electrostatic repulsions between protein building blocks allowing increased ordering of the N-terminal RNA-binding domain. In both those cases the PSs form stem-loops in contrast to the dsRNA regions of STMV. In each case, however, PS-RNA interactions bias assembly towards a subset of the possible assembly pathways due to differential PS-CP affinities [18]. Specific PS binding moreover enhances assembly efficiency by triggering a collapse in the hydrodynamic radius of the genome below the inner radius of the virus protein shell [52], enabling the assembly of the protein shell around the compacted genome.

Knowledge of the precise locations of the PSs and connectivity between them, which is provided by the analysis presented here, is therefore an important component in understanding the mechanisms by which viruses achieve the observed assembly fidelity and efficiency *in vivo*. This, in turn, is a prerequisite for the development of novel antiviral strategies that target virus assembly. As demonstrated in [18], drugs interrupting PS-CP interactions can slow down the assembly process and decrease viral yield via misencapsidation of cellular RNAs. Moreover, a better understanding of conserved features in the genome organization within a viral family provides novel insights into the selective pressures on viral evolution. The method described here enables the identification of such features, and therefore also has profound implications for our understanding of viral evolution.

## Methods

### Asymmetrically averaged structure

The analysis was based on an asymmetric averaged tomogram of MS2 (Fig. 1C) [6], obtained by imaging mature MS2 bound to its natural receptor, the F-pilus of *E*. *coli*. A total of 22 tomograms were taken with 2374 bound viral particles. The 1500 best correlating virion subtomograms (63% of the total) were normalized, low-pass Fourier filtered to 30Å, and then averaged to produce a structure at 39Å resolution. The data was presented as a density map of 64^3^ pixels, sampled to 9.12Å per pixel (EMD-2365).

### Difference map between tomogram and X-ray protein structure

A difference map between the asymmetric EM reconstruction [6] and the X-ray structure of the protein capsid (PDBID 2MS2) was determined as follows: the protein structure was filtered to 39Å resolution to match the EM data; then the pixel size and orientation of the two maps were made equivalent by trilinear interpolation of the reduced-resolution X-ray structure with Chimera [53]. Radial plots compared the distribution of density in the protein map and the tomogram, with the pilus/MP complex masked away for the calculation. The radial distributions were, as expected, similar in the radial ranges corresponding to CP, but different elsewhere at radial levels corresponding to viral RNA (which is organized as a two-shell architecture, see [11]) and the 44kDa single-copy MP. Note that the radial distributions were not identical in the area overlapping with CP—this was due to the low resolution of the map, as CP density could not easily be accounted for in the asymmetric map. Therefore, a contour mask of the tomogram with the protein map was used to sample the low-resolution map, and used to eliminate the protein density via the UCSF Chimera mask routine [53], rather than a direct subtraction of the normalized maps. A mask of 0.5σ best isolated the RNA whilst excluding protein. Finally, two icosahedral masks were applied: the inner core of RNA was masked away under radius 80Å, and an outside mask of radius 120Å removed noise resulting from masking artifacts and the pilus/MP complex. The resultant pruned density contained information about (i) the outer RNA shell in contact with CP, (ii) MP, and (iii) potential traces of CP lying within the shell that were not captured by the masking process.

### Difference map between icosahedrally-averaged EM density and the asymmetric structure

A difference map was created between the icosahedrally-averaged map [11] and the asymmetric structure [6]. We based our analysis on density map EMD-1431 of mature MS2, which was calculated using single particle analysis of 9,335 separate images, equating to ∼560,000 sample points with icosahedral averaging [11]. We used a procedure analogous to the one described above for the tomogram to isolate the RNA. The protein structure was filtered to 9.5Å resolution, with a grid spacing of 1.26Å, to match the symmetric map, and normalization of the resultant protein map to the CP area of the symmetric map was performed. The resampled filtered protein was then subtracted from the symmetric map, yielding a symmetric cage of RNA with a polyhedral shape as in Fig. 1B. The outer shell of RNA was isolated by icosahedral masking with vertex radii of 80Å and 120Å. The resulting map for the outer RNA shell in the icosahedrally-averaged map was aligned with that for the asymmetric RNA organization in the tomogram by reference to the X-ray protein structure used to create each difference map, via UCSF Chimera [53]. After normalization, the aligned maps had similar average, standard deviation and maximum density values.

### Mapping data onto the geometric model

The UCSF Chimera Segment Map tool [54] was used to perform a (watershed) segmentation on the symmetric RNA cage density, which partitioned the polyhedral density into segments attributed to its edges. Each long edge of the cage in Fig. 1B was represented by three segments as shown in S2 Fig. The same watershed segmentation was applied to the asymmetric RNA outer shell map. Hence pixels from the asymmetric RNA map were associated with defined segments on the polyhedral shell, and each connection thus had a density profile associated with it.

We decided to make a very conservative decision on how much data to include, and thus only used the density encoded by the middle segment to represent a long edge. This was because the short segments close to the polyhedral vertices, as well as the short edges themselves, might have contained density corresponding to the RNA-CP contact (i.e. PS) located at a polyhedral vertex bordering the edge, which could have distorted the analysis. Moreover, connections between PS positions adjacent to the MP/pilus (see S1 Fig) were discarded as they may have contained unmasked MP density.

### The density profiles of the long edges

To determine which long edges were occupied, we analysed the density distributions as follows. We computed fitted normal distributions using the *norm*.*fit* function from the *scipy*.*stats* python library, since for a sparse dataset the mean of a fitted normal distribution is less affected by outliers than the raw data. The normal fitting function automatically calculated the best positioning of a unimodal normal distribution for the dataset. Connections occupied in the RNA density were expected to have a substantially higher mean density than unoccupied connections.

Moreover, skews of the distributions were computed via *scipy*.*stats*.*skew*. If a distribution representing density for a connection was negatively skewed, it could not be unambiguously classified as occupied or non-occupied, as this suggested smearing of density. We therefore did not place any constraints on edges with negatively skewed distributions.

## Supporting Information

S1 Fig Positions of long edges omitted from the analysis. Five long edge connections, shown as solid lines between five-fold vertices, are omitted as their proximity to MP makes association of a corresponding RNA density distribution ambiguous.
(TIFF)

S2 Fig Illustration of the segmentation procedure. Each long edge of the polyhedral density (corresponding to the icosahedrally-averaged map) is partitioned into three segments via the UCSF Chimera SegmentMap tool [53, 54]. Only tomographic density overlapping the middle segment (pink) is retained for analysis, as density overlapping with the outer segments (red) may potentially also sample density associated with short edges and RNA-CP connections (i.e. PSs). The segments shown in this figure are from a representative single connection (coloured cyan), not an average of all the connections, and are shown viewed from inside the virion along a particle two-fold axis. CP in the background is shown in beige.
(TIFF)
