# Supplementary figures and images for: Asymmetric Genome Organization in an RNA Virus Revealed via Graph-Theoretical Analysis of Tomographic Data
Source: PLoS Comput Biol. 2015 Mar 20;11(3):e1004146. doi: 10.1371/journal.pcbi.1004146 (PMC4368512; doi:10.1371/journal.pcbi.1004146)

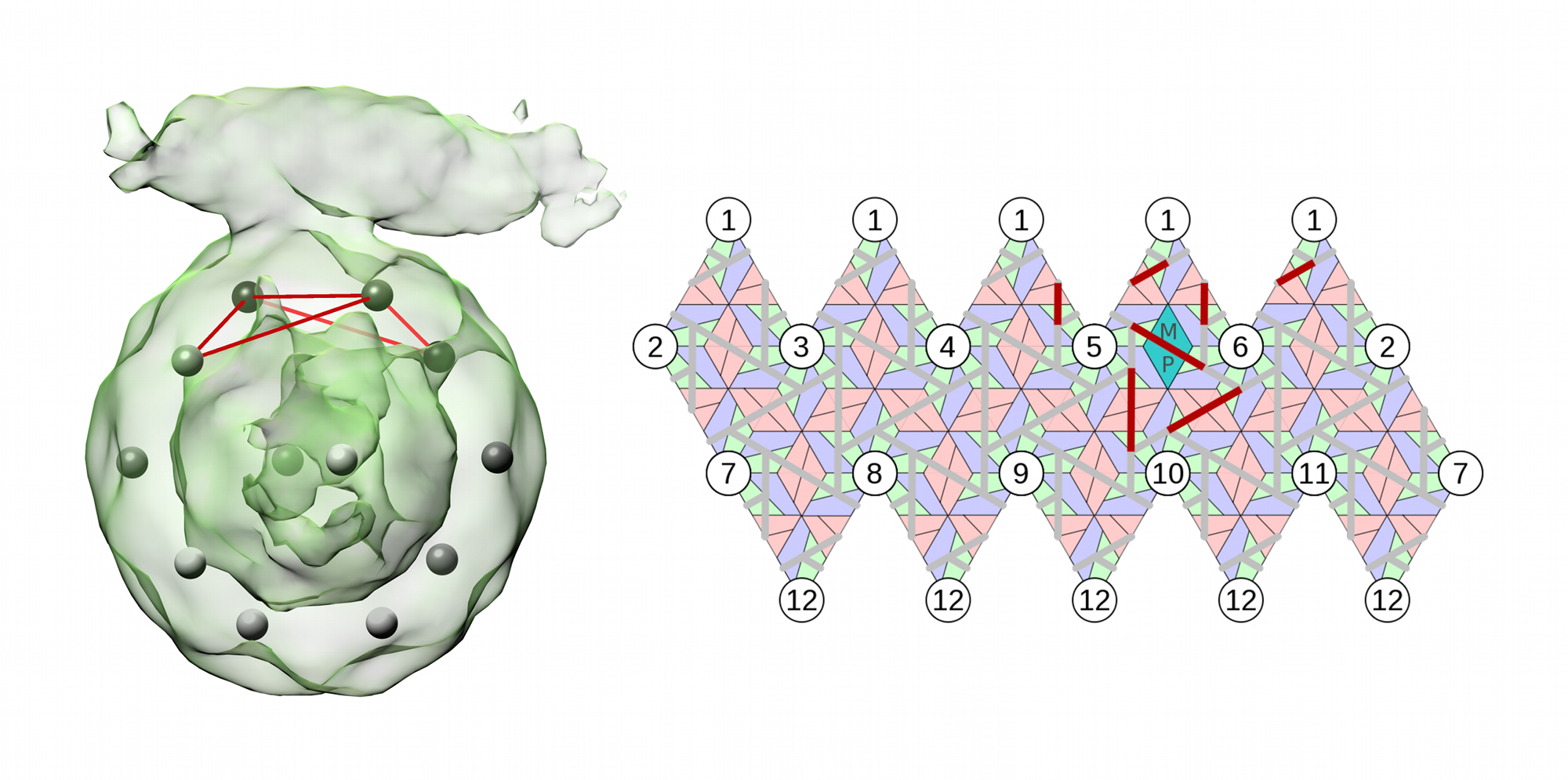

Supplement: S1 Fig — Five long edge connections, shown as solid lines between five-fold vertices, are omitted as their proximity to MP makes association of a corresponding RNA density distribution ambiguous. (TIFF) [file pcbi.1004146.s001.tiff]

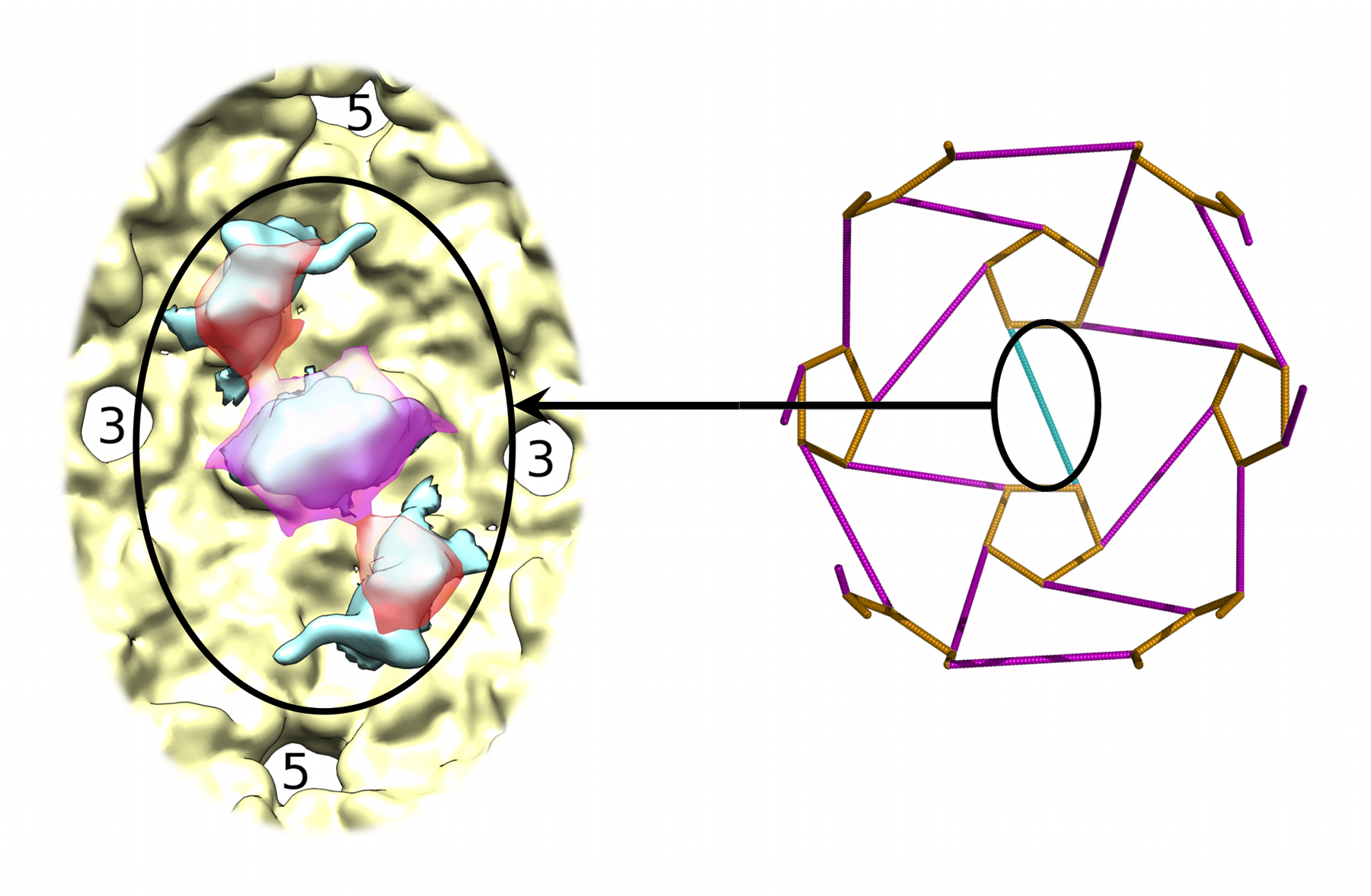

Supplement: S2 Fig — Each long edge of the polyhedral density (corresponding to the icosahedrally-averaged map) is partitioned into three segments via the UCSF Chimera SegmentMap tool [53, 54]. Only tomographic density overlapping the middle segment (pink) is retained for analysis, as density overlapping with the outer segments (red) may potentially also sample density associated with short edges and RNA-CP connections (i.e. PSs). The segments shown in this figure are from a representative single connection (coloured cyan), not an average of all the connections, and are shown viewed from inside the virion along a particle two-fold axis. CP in the background is shown in beige. (TIFF) [file pcbi.1004146.s002.tiff]
